# Molecular Epidemiology of Amoebiasis: A Cross-Sectional Study among North East Indian Population

**DOI:** 10.1371/journal.pntd.0004225

**Published:** 2015-12-03

**Authors:** Joyobrato Nath, Sankar Kumar Ghosh, Baby Singha, Jaishree Paul

**Affiliations:** 1 Department of Zoology, Gurucharan College, Silchar, Assam, India; 2 Department of Biotechnology, Assam University, Silchar, Assam, India; 3 School of Life Sciences, Jawaharlal Nehru University, New Delhi, India; International Centre for Diarrhoeal Disease Research, BANGLADESH

## Abstract

**Background:**

Epidemiological studies carried out using culture or microscopy in most of the amoebiasis endemic developing countries, yielded confusing results since none of these could differentiate the pathogenic *Entamoeba histolytica* from the non-pathogenic *Entamoeba dispar* and *Entamoeba moshkovskii*. The Northeastern part of India is a hot spot of infection since the climatic conditions are most conducive for the infection and so far no systemic study has been carried out in this region.

**Methodology/Principal Findings:**

Following a cross-sectional study designed during the period 2011–2014, a total of 1260 fecal samples collected from the Northeast Indian population were subjected to microscopy, fecal culture and a sensitive and specific DNA dot blot screening assay developed in our laboratory targeting the *Entamoeba* spp. Further species discrimination using PCR assay performed in microscopy, culture and DNA dot blot screening positive samples showed *E*. *histolytica* an overall prevalence rate of 11.1%, 8.0% and 13.7% respectively. In addition, infection rates of nonpathogenic *E*. *dispar* and *E*. *moshkovskii* were 11.8% (95% CI = 10.2, 13.8) and 7.8% (95% CI = 6.4, 9.4) respectively. The spatial distributions of infection were 18.2% (107/588) of Assam, 11.7% (23/197) of Manipur, 10.2% (21/207) of Meghalaya, and 8.2% (22/268) of Tripura states. Association study of the disease with demographic features suggested poor living condition (OR = 3.21; 95% CI = 1.83, 5.63), previous history of infection in family member (OR = 3.18; 95% CI = 2.09, 4.82) and unhygienic toilet facility (OR = 1.79; 95% CI = 1.28, 2.49) as significant risk factors for amoebiasis. Children in age group <15 yr, participants having lower levels of education, and daily laborers exhibited a higher infection rate.

**Conclusions/Significance:**

Despite the importance of molecular diagnosis of amoebiasis, molecular epidemiological data based on a large sample size from endemic countries are rarely reported in the literature. Improved and faster method of diagnosis employed here to dissect out the pathogenic from the nonpathogenic species would help the clinicians to prescribe the appropriate anti-amoebic drug.

## Author Summary

Most epidemiologic studies in developing countries carried out for amoebiasis is either based on microscopy alone or culture/ microscopy used as a screening tool, have poor sensitivity and specificity and thus fails to figure out its true magnitude. The purpose of this study was to assess the true prevalence of amoebiasis in selected North Eastern states of India using DNA based screening technique followed by PCR assay for species discrimination. In addition, PCR assay confirmed that only 55.8% of the samples, resembling *E*. *histolytica* by microscopy, were true *E*. *histolytica*, implying that remaining 44.2% of so-called infections were due to other nonpathogenic *Entamoeba* spp. We found a higher prevalence of amebiasis (13.7%) using DNA dot blot screening compared to conventional microscopy and culture based screening. Poor living condition, previous history of infection in a family member, unhygienic toilet facility, children in age group <15 yr, participants having lower levels of education and daily laborers were identified as significant risk factors for amoebiasis. Thus, the techniques like DNA dot blot hybridization and PCR based detection adopted in the present study over and above the conventional screening methods can reduce misdiagnosis of the disease appreciably from the population living in this endemic area.

## Introduction

Amoebiasis, an infection by protozoa *E*. *histolytica* is appraised as the third leading parasitic cause of human mortality after malaria and schistosomiasis, causing 40 thousand to 100 thousand deaths annually [1]. The re-classification of *E*. *histolytica* into *Entamoeba* complex comprising pathogenic *E*. *histolytica* and nonpathogenic *E*. *dispar* and *E*. *moshkovskii* has further added to the complexity of amoebiasis diagnosis and epidemiology.

Fecal microscopy, the most commonly used clinical diagnostic used for ages; particularly in resource-limited settings are unable to differentiate these three species except in rare invasive cases where fecal samples frequently found to contain hematophagous trophozoites. It was estimated that on an average only 1% of total *E*. *histolytica* infections develop into invasive form and rest remain asymptomatic [2]. Likewise, stool culture based diagnostic methods are time-consuming, laborious and often unrewarding, with a sensitivity of only about 50% [3]. Beside microscopy and stool culture, commercial ELISA based method is among the various other approaches followed for specific identification and detection of *E*. *histolytica* in fecal specimens [4–6]. However, few studies while diagnosing the parasite directly from the stool samples have shown poor sensitivity and specificity due to cross contaminations with other parasites. [7,8].

A number of polymerase chain reaction based assays have been developed over the years; mostly targeting unique regions of the SSU rRNA, as its high copy number provides increased sensitivity [9–12]. However, since the technique could not be made cost effective, therefore, till today prevalence rate reported from developing countries is either based on microscopy alone or molecular assay performed on culture/ microscopy screened samples which themselves have low sensitivities [12–18]. Thus, so far as epidemiology of amoebiasis is concerned, there is a paucity of available documented figure describing its true magnitude particularly from developing countries including India. In line with this, very little is known about the molecular epidemiology of amoebiasis in North Eastern population of India. The aim of the present study was to assess the epidemiologic picture of amoebiasis in selected North Eastern states of India during the 3 year period (2011–2014) using a sensitive and systematic protocol developed in our previous study [19].

## Materials and Methods

### Study design, area and period

A comparative cross-sectional study based on a single fecal sample per person was conducted to figure out the true prevalence of amoebiasis from January 2011 to January 2014. The study was carried out in four selected North Eastern states of India (Assam, Manipur, Meghalaya and Tripura) at the levels of community, healthcare facilities and hospitals.

### Ethics statement

After explaining the importance, purpose and procedure of the study, informed consents were obtained from study participants. For children aged 1 to 10 years consent was systematically sought from the family heads or guardians. Prior to our study, the study protocol was reviewed and approved by the Institutional Ethical Committee (IEC) of Gurucharan College, Silchar, Assam and Assam University, Silchar, Assam, India (IEC/AUS/2013-006).

### Sampling and conventional screening

About 5g of fresh fecal samples were collected in a pre-labelled, clean wide mouth screw-capped container. The samples were collected on the following day within 2–3 h of defecation and delivered to the laboratory and divided into aliquots. One aliquot of each of the fecal samples was used immediately for direct microscopy and inoculated for the establishment of a culture. A part of remaining aliquot was stored at 4°C for formal ether concentration of cysts (for screening by DNA dot blot hybridization), and the third aliquot is stored at -20°C for PCR assay. Samples from distant areas were collected in duplicate. One aliquot was preserved in 10% aqueous formalin for microscopy upon arrival in the laboratory. The other aliquot of the sample was inoculated in culture medium on the spot, and the rest was brought to the laboratory in unpreserved condition by maintaining temperature of approximately 4°C.

Iodine wet mounts of fresh unpreserved fecal samples were examined microscopically for demonstrating cysts and trophozoites of *Entamoeba* species complex. Briefly, a small fraction of feces was mixed with a small drop of Lugols iodine (diluted 1: 5 with water) on a microscope slide, and observed under microscope after placing a cover slip over the preparation. Irrespective of the microscopic analysis results, all fecal samples were cultured for *Entamoeba* species under xenic condition using biphasic (solid and liquid) Robinson’s medium within 5–6 h of collection as previously described [20]. The presence of characteristic spherical, oval or round shaped quadrinucleated cyst or trophozoites in fecal sample; and trophozoites emerging out of excysted cysts with ingested starch particles in xenic culture often showing clear pseudopodia were considered as the keys to confirm sample as positive microscopically. The culture, showing excysted cysts into trophozoites was further subcultured in biphasic Robinson’s medium and after 3–5 passages; the culture was expanded to increase the number of cells for isolation of genomic DNA.

Data on selected independent variables were collected by interviewing all the subjects using pre-designed questionnaire which consists of three sections: 1) General socio-demographic data: age, gender, residence, education, marital status, income and occupation, etc. 2) Environmental factors: toilet facility, water supply, animal contact, contacts with animal feces, etc. 3) Clinical information: anti-amoebic treatment taken previously, previous history of infection, symptomatic (stomach cramping, presence of mucus and blood in stool etc.) or asymptomatic at the time of sample collection etc.

### Dot blot hybridization

DNA dot blot hybridization was performed for screening out the *Entamoeba* (*Entamoeba histolytica* and *Entamoeba dispar*) positive samples. The probe used for the purpose was HMe probe (EcoRI+ Hind III) as previously published [21]. Briefly, crude DNA was obtained from enriched cysts from stool samples directly by five freeze-thaw cycles followed by sonication. After denaturing crude cyst DNA using NaOH to a final concentration of 0.25 N, the DNA was spotted in triplicate on to the GS+ nylon membrane pre-saturated in 0.4 M Tris-Cl, pH 7.5 with the help of mini-fold apparatus. The air-dried and UV cross-linked blots were then ready for hybridization with 4.5 kb rDNA fragment (EcoRI—Hind III) from HMe region of EhR1 (rDNA plasmid in HM1: IMSS strain of *E*. *histolytica*).

### Extraction of genomic DNA

Genomic DNA was extracted from an aliquot of 200 mg fecal sample using a DNA stool kit (Qiagen, Valencia, CA). Briefly, with the addition of five freezing-thawing cycles, samples were vortexed vigorously for 5–10 minutes in lysis buffer (ASL buffer). The samples were then processed according to the instructions of the manufacturer with slight variations, particularly incubation of the DNA in the spin column in elution buffer was carried out for 3 minutes at room temperature followed by centrifugation and this final elution step was repeated twice using 25 μl elution buffer each time to increase the DNA yield. The DNA was then stored at −20°C until used for PCR amplification.

For isolation of genomic DNA from cultured cell, trophozoites from the positive culture medium were harvested from 6–8 fully-grown culture tubes by chilling, followed by centrifugation at 600g for 5 minutes at 4°C. The cell pellet was then washed twice with 20 ml of PBS and finally stored in 70% ethanol at -20°C. The cells were pelleted through centrifugation at 13000 rpm for 4 min; air-dried to remove all traces of ethanol. The DNA was then isolated from pelleted cells using Genomic DNA mini kit (Real Genomics, Taiwan) following manufacturer’s instructions and finally eluted in 30–50 μl of elution buffer.

### Discrimination of *E*. *histolytica*, *E*. *dispar* and *E*. *moshkovskii*

Forward and reverse oligonucleotide primers targeting the signature sequence of the infecting parasite were used for PCR assay. Amplification for *E*. *histolytica* was achieved using a nested PCR protocol with primer set E-1: 5^/^ TAA GAT GCA CGA GAG CGA AA 3^/^ and E-2: 5^/^ GTA CAA AGG GCA GGG ACG TA 3^/^ for primary PCR and primer set EH-1: 5^/^- AAG CAT TGT TTC TAG ATC TGA G-3^/^) and EH-2 (5^/^- AAG AGG TCT AAC CGA AAT TAG- 3^/^) for secondary PCR [22]. A common forward primer sequence EntaF was used for amplifying *E*. *dispar* and *E*. *moshkovskii*, whereas EdR and EmR were used as species-specific reverse primer for distinguishing the two species. Primer sequences used were as follows: EntaF: 5^’^-ATG CAC GAG AGC GAA AGC AT-3’ and EdR: 5^’^-CACCACTTACTATCCCTACC-3^’^) to detect *E*. *dispar*; EntaF: 5´-ATG CAC GAG AGC GAA AGC AT- 3´ EmR: 5´-TGA CCG GAG CCA GAG ACA T-3´to detect *E*. *moshkovskii* [23]. Briefly, all the PCR amplifications were performed in a final volume of 20μl with approximately 100ng of template DNA, 1 μM of each primer, 1X PCR buffer with 2.5 mM MgCl_2_, 1X BSA, 0.2 mM dNTPs, and 1U of Taq DNA Polymerase (Thermo scientific, Wattham, USA) in the thermal cycler (Bio-Rad Laboratories, Hercules, CA). Parasitic infection was confirmed by their expected amplicon sizes of 439 bp, 752 bp, and 580 bp for *E*. *histolytica*, *E*. *dispar* and *E*. *moshkovskii* respectively through gel electrophoresis. Some of the random signature amplicons were sequenced directly using respective primer pair using ABI 3500 Genetic analyzer (Applied Biosystems Inc., CA, USA) and subjected to homology search using nucleotide blast (blastn) program available at National Centre for Biotechnological Information (http://www.ncbi.nlm.nih.gov) database for further confirmation.

### Data analysis

Data entry and statistical analysis were performed with the aid of SPSS statistical software version 16.0 (SPSS, Chicago, IL, USA) and MedCalc version 15.4 (MedCalc Software bvba, Belgium). Categorical variables were described using numbers and percentages. Descriptive statistics were used to show any association of disease with the variables like age, sex and others. We used Pearson’s Chi-square test at a level of significance P< 0.05 to test the associations of infection frequencies among groups in univariate statistical model. Frequencies of infection were used as the dependent variables, while the independent variables were environmental, socio-demographic factors and clinical status of participants. Odds ratios (OR) and 95% confidence intervals were computed to measure the strength of association between determinants of parasitic infection and burden of infection.

## Results

### Study compliance

From a total of 1450 study subjects recruited from the 17 selected sites in the North Eastern states of India, 1260 participated in the cross-sectional survey, owing to an overall compliance of 86.9%. Reasons for non-compliance were absence of communication in the following day of sample collection (n = 13), absence of written informed consent/ questionnaire (n = 27), samples not deposited or because of production of insufficient specimens (n = 57) and few withdrew from the study without specific reason (n = 93). In total, 1260 stool samples were finally collected for the study.

Among this 1260 samples, 588 (46.7%) were from the Assam state, 268 (21.3%) from the Tripura state, 207 (16.4%) from the Meghalaya state and 197 (15.6%) from the Manipur state. Samples were collected over a period of three years from 2011–2014 during different seasons of the year, 373 (29.6%) were collected during pre-monsoon (Feb-May) season while 451 (35.8%) and 436 (34.6%) were collected during monsoon (Jun-Sep) and post-monsoon season (Oct-Jan) respectively (S1 Table).

### Microscopy, culture and dot blot screening of fecal samples

**Fig 1:**
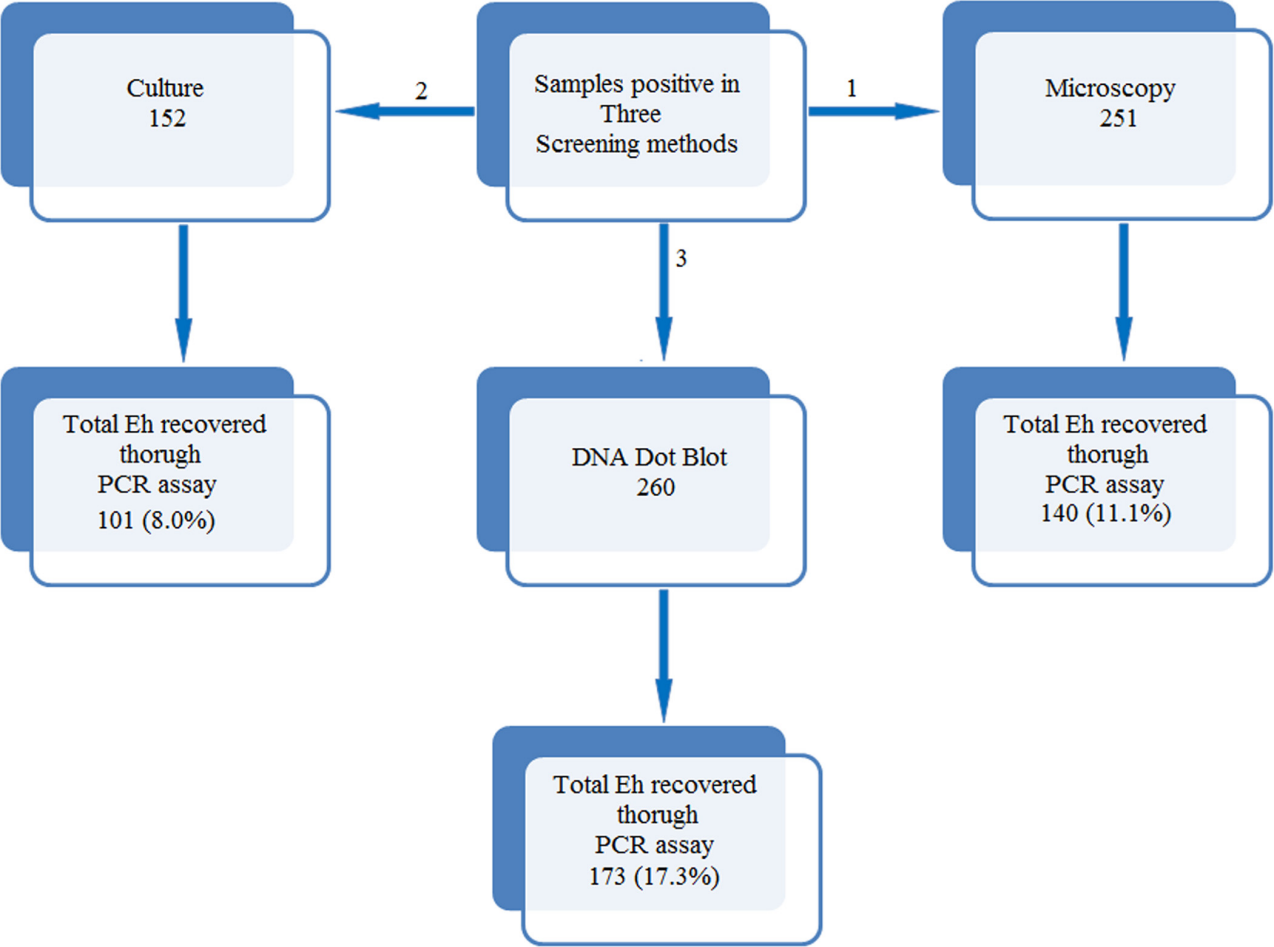
Screening protocol for *Entamoeba* positive stool samples using different screening techniques. Numbers in each box represent positive samples obtained by each method out of total 1260 stool samples. The DNA Dot blot was carried out using a probe that hybridizes with E.h and /or E.d positive DNA samples.

Analysis of the 1260 fecal samples by microscopic examination of a direct saline (wet) mount, *E*. *histolytica*-like cysts or trophozoites were detected in 251 (~19%) samples, whereas, 152 (~12%) samples showed positive result in biphasic xenic culture (Fig 1). Interestingly, when crude DNA extracted from all the fecal samples (1260) were passed through third screening technique, DNA dot blot, 260 (20.6%) showed positive spots. On further analysis of our samples it was observed that amongst the 129 (10.2%) fecal samples that were positive in both microscopy and culture (last row of Table 1) only 118 were positive in DNA dot blot assay while remaining 11 were negative suggesting the false positives associated with conventional assay. Similarly, 122 that were exclusively positive in microscopy and 23 fecal samples that were positive in culture only, dot blot hybridization detected *Entamoeba* complex in 83 and 20 respectively. Thus the remaining 53 samples that were positive in either of the two conventional screening techniques that is the microscopy and culture, but negative in DNA dot blot assay were repeated again for hybridization, but did not yield a positive signal. While, fecal samples in which no cysts and trophozoites stages of *Entamoeba* complex was detected by microscopic and/ xenic culture examination, 39 were identified as positive when screened by dot blot assay suggesting the association of false negatives with conventional screening method. The samples positive using any of these three screening methods were then subjected to species specific singleplex PCR assay on genomic DNA isolated from stool samples directly. Genomic DNA from each species was used as positive controls.

**Table 1:** Species discrimination of samples positive in microscopy, culture and dot blot screening using species specific PCR assay.

Mono and mixed infection as detected in Singleplex PCR assay	Microscopy and culture result				Total no in PCR assay
	Positive by microscopy & culture	Positive by microscopy only	Positive by culture only	Negative by microscopy and culture	
*E*. *histolytica*	37*	19*	9*	11*	76
*E*. *dispar*	29*	21*	4*	20*	74
*E*. *moshkovskii*	4	28	0	0	32
*E*. *moshkovskii* + *E*. *dispar*	4*	7*	0	2*	13
*E*. *histolytica* + *E*. *dispar*	19*	17*	5*	3*	44
*E*. *histolytica* + *E*. *moshkovskii*	18*	13*	1*	3*	35
*E*. *histolytica* + *E*. *dispar* + *E*. *moshkovskii*	11*	6*	1*	0	18
Negative	7	11	3	947	968
**Total**	129 (10.2%)	122 (9.7%)	23 (1.8%)	986 (78.3%)	1260

*Indicates samples positive either for *E*. *histolytica* or *E*. *dispar* or mixed when screened by DNA dot blot hybridization technique. The dot blot screen did not include a probe for *E*. *moshkovskii*.

### Comparison of microscopy, culture and DNA dot blot with PCR assay

PCR amplification targeting signature sequence of small ribosomal RNA gene of *E*. *histolytica*, *E*. *dispar* and *E*. *moshk*ovs*kii* produced diagnostic amplicons of 439 bp, 752 bp, and 580 bp respectively. Amongst 122 fecal samples that were positive only in microscopy using wet preparation, 19 (15.6%) were *E*. *histolytica* (mono-infection) infected, 21 (17.2%) were *E*. *dispar* (mono-infection) infected, 28 (23.0%) were *E*. *moshkovskii* (mono-infection) infected, 7 (5.7%) were infected both with *E*. *dispar* and *E*. *moshkovskii* (mixed-infection) and 36 (29.5%) were mixed infections of *E*. *histolytica* with either *E*. *dispar* or *E*. *moshkovskii* or all the three species, while 11 (9.0%) were negative in all the three PCR assays (Table 1). Similarly, when PCR was performed on 23 fecal samples that were positive by culture only, we failed to amplify the signature location in DNA isolated from 3 (13.0%) samples while among the remaining 20 samples *E*. *histolytica* (mono-infection) infections were found in 9 (39.1%), *E*. *dispar* (mono-infection) infections were found in 4 (17.4%) and mixed infections of *E*. *histolytica* with either *E*. *dispar* or *E*. *moshkovskii* or both were found in 7 (30.4%) samples. Among the 260 dot blot positive samples, mono-infections of *E*. *histolytica* and *E*. *dispar* were detected in 111 (42.7%) and 87 (33.5%) respectively, and mixed infections of both in 62 (23.8%) samples. Thus, comparison of molecular technique with classical techniques (microscopy and culture based) revealed that the DNA dot blot hybridization technique followed by validation with PCR is necessary to arrive at the true prevalence of *E*. *histolytica* among the study population. The detailed analysis of various screening techniques and their outcome is represented in the Fig 1.

### Prevalence of *E*. *histolytica*, *E*. *dispar* and *E*. *moshkovskii*

The overall prevalence of any of the three morphologically indistinguishable *Entamoeba* species (pathogenic and non-pathogenic) was 23.2% (95% CI = 20.9%, 25.6%). Table 2 shows that 13.7% (173/1260; 95% CI = 11.9, 15.7) and 11.8% (149/1260; 95% CI = 10.2, 13.8) of the subjects were infected with *E*. *histolytica* and *E*. *dispar*, respectively. This 13.7% of the total samples (1260) were positive in the PCR assay either singly for *E*. *histolytica* or in combination with other intestinal protozoan parasites. Clinical specimens such as fecal sample often contain PCR inhibitors even after purification steps during genomic DNA isolation. In order to rule out this possibility, 21 culture and/ or microscopically positive, but PCR negative samples were further seeded with control DNA of HM1: IMSS strain of *E*. *histolytica*. In all the cases spiking with control DNA yielded a positive amplification suggesting that this 21 microscopy and/ or culture positive samples were actually false positive and thus negative results obtained in PCR assay is not because of PCR inhibitors.

**Table 2:** Prevalence rate; mono and mixed infection of *E*. *histolytica*, *E*. *dispar* and *E*. *moshkovskii* as scored by species specific PCR assay.

*Entamoeba* species (mono and mixed infections)	Total No. positive	Total EH* (Prevalence)	Total ED* (Prevalence)	Total EM* (Prevalence)
*E*. *histolytica* (mono-infection)	76	76	-	-
*E*. *dispar* (mono-infection)	74	-	74	-
*E*. *moshkovskii* (mono-infection)	32	-	-	32
*E*. *moshkovskii +E*. *dispar* (mixed-infection)	13	-	13	13
*E*. *histolytica + E*. *dispar* (mixed-infection)	44	44	44	-
*E*. *histolytica + E*. *moshkovskii* (mixed- infection)	35	35	-	35
*E*. *histolytica + E*. *dispar + E*. *moshkovskii* (mixed-infection)	18	18	18	18
**Total (%)**	292	173 (13.7)	149 (11.8)	98 (7.8)

*EH = *E*. *histolytica*

*ED = *E*. *dispar*

*EM = *E*. *moshkovskii*

**Fig 2:**
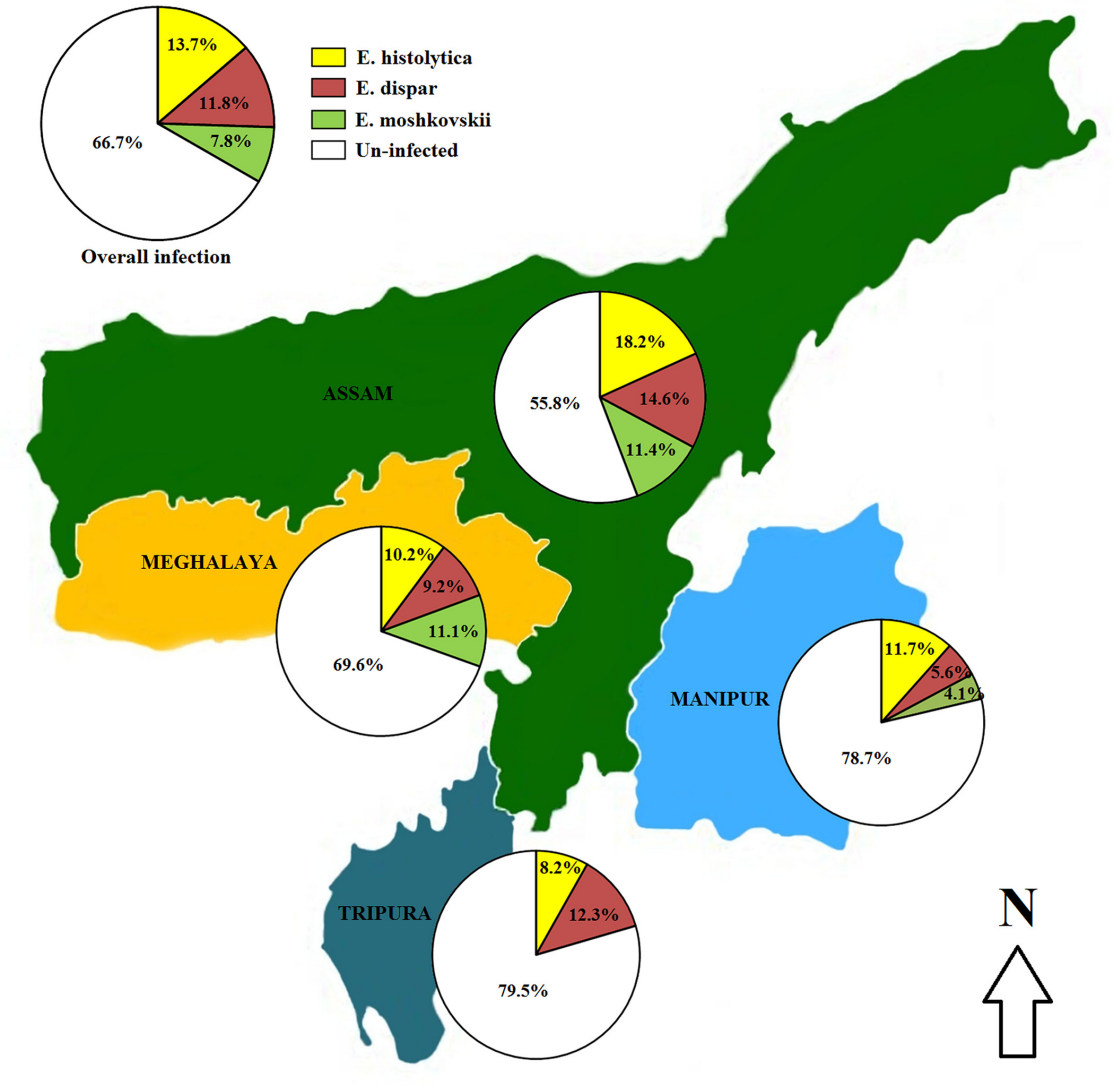
Prevalence of *E*. *histolytica*, *E*. *dispar* and *E*. *moshkovskii* stratified by four Northeast Indian states.

Among the 1260 fecal samples collected from the four North Eastern states viz., Assam, Meghalaya, Manipur and Tripura at the level of community health care units and hospitals, highest *E*. *histolytica* prevalence was recorded in Assam 18.2% (95% CI = 15.2, 21.6), followed by 11.7% (95% CI = 7.7, 17.2) in Manipur, 10.2% (95% CI = 6.5, 15.3) in Meghalaya, while 8.2% (95% CI = 5.3, 12.3) in Tripura had the least (Table 3). Prevalence of *E*. *dispar* was highest with 14.6% (95% CI = 11.9, 17.8) in Assam followed by 12.3% (95% CI = 8.7, 17.0) in Tripura, 9.2% (95% CI = 5.8, 14.2) in Meghalaya and 5.6% (95% CI = 3.0, 10.0) in Manipur. Prevalence of *E*. *moshkovskii* was almost equal in Assam with 11.4% (95% CI = 9.0, 14.3) and the Meghalaya state with 11.1% (95% CI = 7.3, 16.4) (Fig 2).

**Table 3:** Prevalence of *E*. *histolytica*, *E*. *dispar* and *E*. *moshkovskii* infection, stratified by four states of North East under study during January, 2011 to January, 2014.

State	No. examined	EH* Infection	Proportion (95% CI)	ED* Infection	Proportion (95% CI)	EM* Infection	Proportion (95% CI)
**Assam**	588	107	18.2	86	14.6	67	11.4
			(15.2, 21.6)		(11.9, 17.8)		(9.0, 14.3)
**Meghalaya**	207	21	10.2	19	9.2	23	11.1
			(6.5, 15.3)		(5.8, 14.2)		(7.3, 16.4)
**Manipur**	197	23	11.7	11	5.6	8	4.1
			(7.7, 17.2)		(3.0, 10.0)		(1.9, 8.3)
**Tripura**	268	22	8.2	33	12.3	0	0
			(5.3, 12.3)		(8.7, 17.0)		
**Total**	1260	173	13.7	149	11.8	98	7.8
			(11.9, 15.7)		(10.2, 13.8)		(6.4, 9.4)

*EH = *E*. *histolytica*

*ED = *E*. *dispar*

*EM = *E*. *moshkovskii*

### Socio-demographic characteristics and prevalence of *E*. *histolytica* infection

Univariate analysis of demographics and the prevalences of *E*. *histolytica* infection were presented in Table 4. The prevalence showed an age dependency association, with significantly higher infection rates among respondents aged less than 15 years (OR = 3.06; 95% CI = 1.90, 4.94; P< 0.001) and in the age group 15–30 (OR = 2.36; 95% CI = 1.40, 3.97; P< 0.001). It was observed that the prevalence rate decreased from 17.6% to 8.9%, with higher education level of the participants (OR = 2.18; 95% CI = 1.31, 3.63; P = 0.003). Six hundred eighty one (54.0%) of the participants were from rural areas. Infection was higher among respondents from the rural population (OR = 1.62; 95% CI = 1.16, 2.27; P = 0.004) than those from urban population.

**Table 4:** Socio-demographic features of the study participants and their association with *E*. *histolytica* infection.

Variables	No. examined	No. Positive (%)	OR (95% CI)	P value
**Occupation**				**0.005**
Government employees	198	17 (8.6)	1*	
Student & pre-school	398	71 (17.8)	2.31 (1.32, 4.05)	
Merchant	212	21 (9.9)	1.17 (0.60, 2.29)	
Daily laborers including Farmer, driver	231	38 (16.5)	2.10 (1.14, 3.85)	
House wife	221	26 (11.8)	1.42 (0.75, 2.70)	
**Education**				**0.003**
Illiterate	346	61 (17.6)	2.18 (1.31, 3.63)	
Primary education	353	57 (16.1)	1.96 (1.17, 3.27)	
High School	304	32 (10.5)	1.20 (0.68, 2.10)	
College and above	257	23 (8.9)	1*	
**Residence**				**0.004**
Rural	681	111 (16.3)	1.62 (1.16, 2.27)	
Urban	579	62 (10.7)	1*	
**Marital status**				0.232
Single	737	94 (12.8)	1*	
Married	523	79 (15.1)	1.23 (0.88, 1.68)	
**Age groups**				**<0.001**
<15	327	64 (19.6)	3.06 (1.90, 4.94)	
15–30	247	39 (15.8)	2.36 (1.40, 3.97)	
31–45	367	27 (7.4)	1*	
>45	319	43 (13.5)	1.96 (1.18, 3.26)	
**Sex**				0.060
Male	497	57 (11.5)	1*	
Female	763	116 (15.2)	1.41 (1.00, 1.98)	
**Income (per day)**				0.336
<500	637	95 (14.9)	1.43 (0.89, 2.31)	
500–1000	413	55 (13.3)	1.23 (0.74, 2.10)	
>1000	210	23 (10.9)	1*	
**Household members**				0.061
>5	331	33 (10.0)	1*	
5–7	521	81 (15.5)	1.66 (1.08, 2.56)	
Above 7	408	59 (14.5)	1.09 (0.76, 1.57)	
**Seasonal prevalence**				**<0.001**
Pre-monsoon (Feb-May)	373	41 (11.0)	1.29 (0.81, 2.059)	
Monsoon (Jun-Sep)	451	94 (20.8)	2.78 (1.84, 4.13)	
Post-monsoon (Oct-Jan)	436	38 (8.7)	1*	

**Fig 3:**
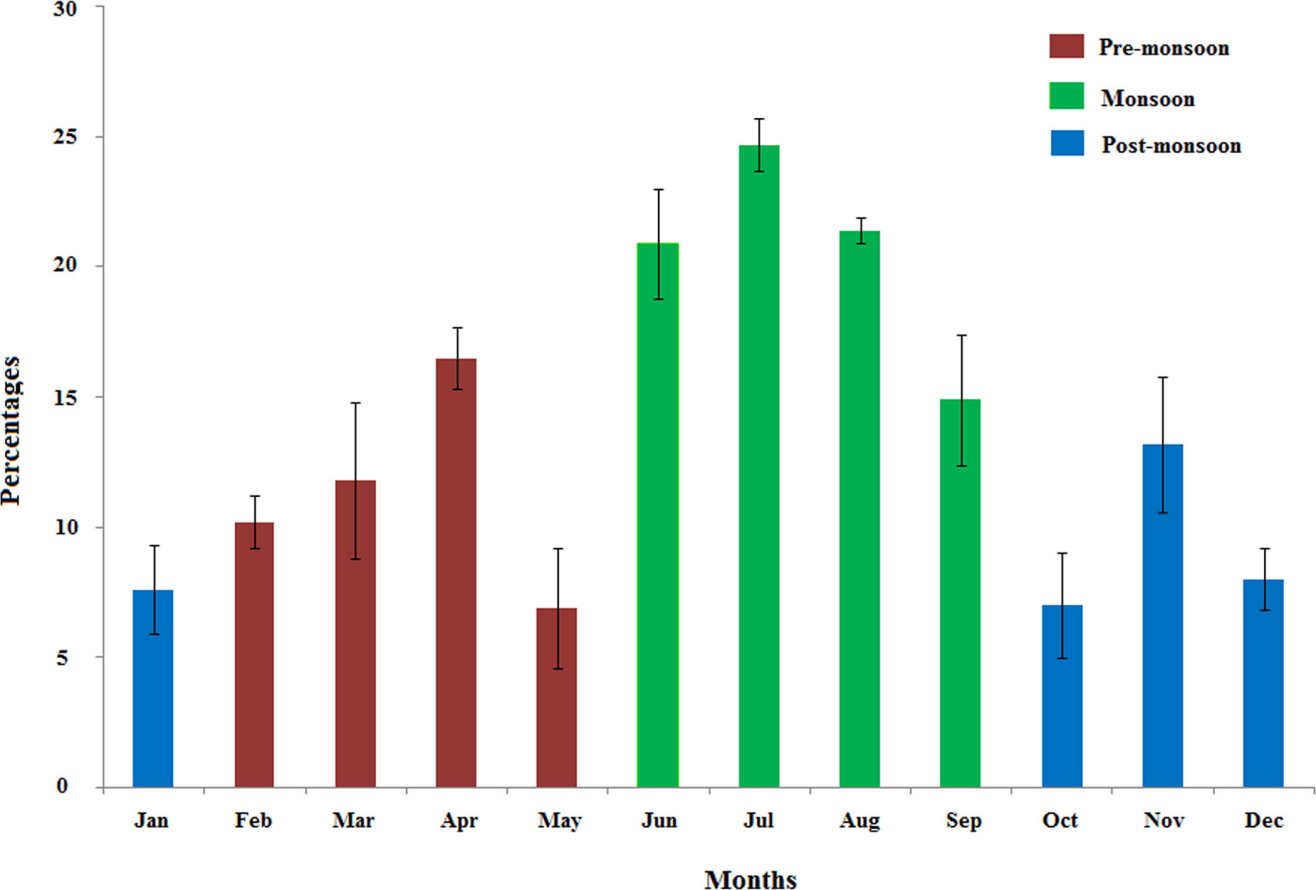
Seasonal variation pattern of *E*. *histolytica* infection rate from January 2011 to January 2014. Percentage values are averaged for each month over a period of three years.

Marital status and gender bias was not significantly associated with the prevalence of *E*. *histolytica* infection, although female (15.2%) had slightly higher prevalence rate compared to male (11.5%). Among other socio-demographic factors, as in various occupational groups, the school students (OR = 2.31; 95% CI = 1.32, 4.05; P = 0·005), followed by truck drivers (OR = 2.10; 95% CI = 1.14, 3.85) and the merchant group (OR = 1.17; 95% CI = 0.60, 2.29) were at higher risk compared to the public service employee group. In context to the seasonal impact on the prevalence, as expected, we observed a significant higher infection rate during the monsoon season (June–September), approximately 21% (OR = 2.78; 95% CI = 1.84, 4.13; P< 0.001) compared to the pre- or post-monsoon seasons. A month wise variation pattern of prevalence rate was shown in Fig 3. Further univariate analysis of the socio-demographic factors showed that the infection was independent of per day income, marital status, gender and family size.

### Association of amoebiasis with selected environmental factors and infection history of participants

Table 5 showed the results of regression analysis of various potential factors associated with *E*. *histolytica* infection rate. We observed participants having unhygienic toilet facility more likely to be infected with *E*. *histolytica* compared to those having hygienic toilet facilities (OR = 1.79; 95% CI = 1.28, 2.49; P = 0.001). In terms of percentage value, participants with a family history of gastrointestinal infection and those have taken anti-amoebic treatment previously were 3.2 (OR = 3.18; 95% CI = 2.09, 4.82; P< 0.001) and 1.9 times (OR = 1.93; 95% CI = 1.40, 2.67; P< 0.001) more likely to be infected. Similarly, subjects having a previous history of infection in life were 1.4 times more likely to be infected compared to those who were not previously infected (OR = 1.40; 95% CI = 1.02, 1.94; P = 0.038).

**Table 5:** Univariate analysis of selected environmental factors and subject’s infection history with prevalence of amoebiasis.

Variables	No. examined	No. Positive (%)	OR (95% CI)	P value
**Toilet facility**				**0.001**
Yes (Hygienic)	597	61 (10.2)	1*	
No (Unhygienic)	663	112 (16.8)	1.79 (1.28, 2.49)	
**Source of water**				**0.003**
Tap water	616	64 (10.4)	1*	
Well/ Pond/ River	388	66 (17.0)	1.78 (1.22, 2.56)	
Both	256	43 (16.8)	1.74 (1.15, 2.64)	
**Animal contact**				0.149
Yes	491	76 (15.5)	1.27 (0.92, 1.75)	
No	769	97 (12.6)	1*	
**Living condition**				**<0.001**
Poor	408	86 (21.1)	3.21 (1.83, 5.63)	
Medium	644	71 (11.0)	1.49 (0.84, 2.62)	
Good	208	16 (7.7)	1*	
**Eating raw vegetables**				0.071
No	889	112 (12.6)	1*	
Yes	371	61 (16.4)	1.37 (0.97, 1.92)	
**History of infection in family member**				**<0.001**
Yes	807	144 (17.8)	3.18 (2.09, 4.82)	
No	453	29 (6.4)	1*	
**Anti-amoebic treatment taken previously**				**<0.001**
Yes	481	90 (18.7)	1.93 (1.40, 2.67)	
No	779	83 (10.7)	1*	
**Previous history of infection (subject)**				**0.038**
Yes	614	97 (15.8)	1.40 (1.02, 1.94)	
No	646	76 (11.8)	1*	
**Hand washing**				**0.002**
Yes	883	104 (11.8)	1*	
No	377	69 (18.3)	1.68 (1.20, 2.33)	
**Stool contains mucus and/or blood**				**<0.001**
Yes	221	62 (28.1)	3.26 (2.29, 4.64)	
No	1039	111 (10.7)	1*	
**Symptom**				**0.005**
Symptomatic	498	85 (17.1)	1.57 (1.14, 2.18)	
Asymptomatic	762	88 (11.5)	1*	

With respect to behavioral characteristics, participants those directly using river, pond water for daily use were more likely to be infected with *E*. *histolytica* (OR = 1.78; 95% CI = 1.22, 2.56; P = 0.003) compared to the group using tap water as a drinking water source. The odds ratio of *E*. *histolytica* infection in participants who belong to poor quality of living condition is 3.21 times higher than those who live a better quality of living. Infection was higher among participants with clinical signs like stomach pain and cramping, passage of either watery or mucous with bloody stool etc. (OR = 1.57; 95% CI = 1.14, 2.18; P = 0.005). The data confirmed that individuals who were in close contact with domestic animals, i.e., dogs and cats were around 1.3 times (OR = 1.27; 95% CI = 0.92, 1.75; P = 0.149) more likely to be infected with *E*. *histolytica* compared to those who do not keep domestic animals as their pets, however the difference was not statistically significant. Similarly, consumption of raw vegetables was not significantly associated with *E*. *histolytica* infection.

## Discussion

**Fig 4:**
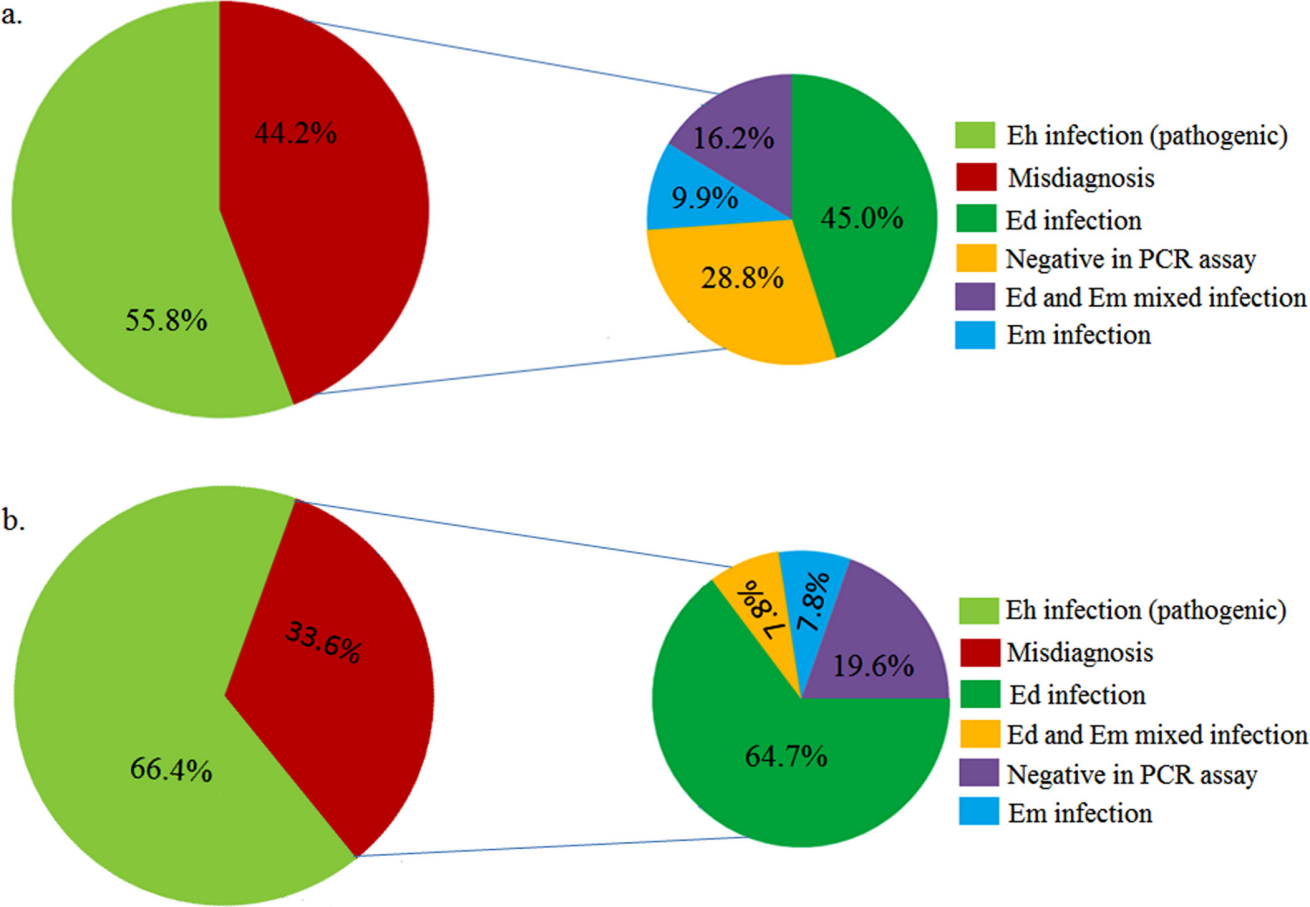
Percentages of misdiagnosis cases associated with conventional diagnostics a) microscopy b) fecal culture. Eh = *E*. *histolytica*, Ed = *E*. *dispar*, Em = *E*. *moshkovskii*.

High rate of parasitic infections encountered in this part of the sub continent and especially the endemic nature of the disease call for improved method of diagnosis. Development of rapid and accurate identification methods are essential for public health efforts to manage the disease. Various techniques such as microscopy, culture, zymodeme analysis, ELISA and DNA based methods are being followed for specific identification of *E*. *histolytica* in fecal specimens. Recent study highlighted the failure of TechLab ELISA kit; in detecting *E*. *histolytica* in some of the *E*. *histolytica* PCR confirmed samples [7,8]. Microscopy has a sensitivity of only 60%, even under optimal standards while fecal culture is less sensitive than microscopy as a detection method [6,22]. In our study, of the 251 samples that were microscopically positive, 56 were *E*. *histolytica* and 84 were mixed infections with *E*. *histolytica*. Thus, only 55.8% of the samples, resembling *E*. *histolytica* by microscopy, were true *E*. *histolytica* as confirmed by PCR assay, implying that remaining 44.2% of so-called infections were due to other two *Entamoeba* spp. (Fig 4). A study conducted among prisoners and primary-school children in Ethiopia highlighted 91.4% of the microscopy positive samples as *E*. *dispar* [24]. In another study reported from Australia, 50% of the microscopy positive fecal samples were found to be positive for nonpathogenic *E*. *moshkovskii* in the PCR assay [25]. The negative PCR result in 18 microscopy positive fecal samples is probably because of the presence of other *Entamoeba* species inhabiting the human gut. However, this needs further confirmation using molecular tools to validate the existence of other commonly found *Entamoeba* species in humans.

As shown by the results of the present study, the three species of *Entamoeba* namely *E*. *histolytica/E*. *dispar/E*. *moshkovskii* are prevalent in North Eastern states of India with an overall prevalence of 23.2%. The prevalence rate of *E*. *histolytica* observed in our cross-sectional study conducted at the community level healthcare unit and hospital using a molecular technique was 13.7%. Because of a better sensitivity of the two molecular methods employed here like DNA dot blot and PCR based methods together, helped us to correctly arrive at the true prevalence of *E*. *histolytica* in the samples collected from this region. This would not have been possible, employing culture and microscopy methods in isolation. Thus the diagnostic sensitivity can be improved by employing above techniques while carrying out epidemiological study in a region particularly endemic for the parasite. According to a recent review 15–20% of the Indian population is affected by *E*. *histolytica* [26]. Studies from different parts of the world indicate variable rate of *E*. *histolytica* prevalence in the fecal samples. A prevalence rate of 13.2% for *E*. *histolytica* and 9.9% for *E*. *dispar* was reported from Orang Asli settlements in Malaysia using real time PCR conducted on microscopy positive samples [27]. A much higher *E*. *histolytica* and *E*. *dispar* prevalence rate of 69.6% and 22.8%, respectively was reported using PCR assay among children in Gaza, Palestine [28]. However, it is very difficult to compare the true prevalence of amoebiasis because of the lack of uniformity in diagnostic methods. Much of the data reported are either based on microscopy alone or PCR assay performed on microscopy screened samples which itself has poor sensitivity. Moreover, it is now well documented that *E*. *dispar* infection is much more prevalent than *E*. *histolytica* worldwide [29,30]. In Agboville town near Abidjan, PCR analysis of microscopically positive samples demonstrated the ratio of *E*. *histolytica* to *E*. *dispar* of 1:46 [31]. Presence of non pathogenic *E*. *moshkovskii* has also been reported from countries like Bangladesh, Turkey, India, Iran, Australia, Tanzania and Malaysia and usually they are not associated with disease [4,13,25,32–34].

Studies from different geographical areas of the globe reported that the intensity of intestinal parasitic infections (IPIs) including *E*. *histolytica* was significantly higher among children [35–37]. However, our results did not show any significant difference in the prevalence of *E*. *histolytica* infection when compared between genders. This supported earlier observations made in different parts of the world [35,38,39]. In contrast, most hospital-based studies reported gender dependent *E*. *histolytica* infection [40–43]. The association between infection and occupational status indicated that student/ pre-school and daily laborers, including farmer, driver were the two groups who presented more than a twofold increased risk compared to Gov’t employers. This could be attributed to the fact that former groups frequently consume street foods not maintained in required hygienic conditions. Further, the participant’s level of education also exhibited significant association with *E*. *histolytica* infection. Rural background of respondents was also significantly associated with *E*. *histolytica* infection. As shown by other previous studies [44,45,46], our study further confirmed a higher risk of *E*. *histolytica* infection among the rural population, where prevailing poverty, no exposure to health education program, poor socioeconomic status, low standards of sanitation and hygiene are the associated factors that contributed to the high rate of infection. As expected, we observed significantly higher infection rate among participants with diarrhea or other gastrointestinal symptoms compared to asymptomatic group. This finding is in parallel with the studies conducted in Malaysia, Turkey, and Sweden [47–49]. A recent review suggested that asymptomatic cyst passage, with 90% of human infections either asymptomatic or mildly symptomatic, is considered to be the most common manifestation of *E*. *histolytica*. However, the above conclusion was based on studies made by fecal microscopy [6]. The risk of harboring the non-pathogenic species cannot be ruled out. In our study, interestingly 7 individuals mono-infected with *E*. *moshkovsk*ii were found to be symptomatic (S1 Table). In a separate study from India and Malaysia, association of *E*. *moshkovskii* infection with dysentery has been reported [13,34]. However, further studies on more samples are necessary to validate the role of *E*. *moshkovskii* in gastroenteritis disorders and its virulence.

Logistic regression analysis indicated that the factors responsible for infection can be pointed to poor living conditions, unhygienic toilet facility, not washing hands before taking food due to which infection rates increased by 3.21, 1.79 and 1.68 fold respectively. Similar risk factors have been described for the infection in population from Italy and Yemen [50,51]. Our data also revealed that the likelihood of acquiring infection due to the parasite among participants who have a record of pervious infection history and those had taken anti-amoebic chemotherapy were 1.4 and 1.9 fold, suggesting the possibility of harboring higher drug tolerant strains among the North Eastern population. However, further studies are warranted, particularly focusing on the metronidazole sensitivity of natural and clinical isolates of *E*. *histolytica*. Our observation of acquiring infection was three times higher in individuals having a history of infection in the family members. This finding was in line with previous studies carried out among the population of El Salvador, Mexican and Orang Asli Ethnic Groups of Malaysia where person-to-person transmission was indicated as the most important determinant of infection [52–54]. Therefore, it is recommended to screen the stool samples of every family member on a routine basis and any person found infected with the pathogenic species should be treated with the antiamebic drug. As expected, we observed highest prevalence of *E*. *histolytica* in the monsoon season followed by the pre- and post-monsoon seasons. This could be attributed to the high rate of fecal–oral contamination during monsoon season.

Our study did not reveal any significant association of *E*. *histolytica* infection with the individuals having close contacts with domestic animals. In contrast to this, reports from countries like Nigeria, Yemen and Malaysia reported an increase in the prevalence of *Entamoeba* complex infection among individuals having close association with domestic animal [55–57]. Recently, *E*. *hartmanni*, *E*. *coli* and *E*. *disp*ar were isolated from captive non-human primates housed in the zoological garden of Rome, highlighting the risk of zoonotic transmission of this parasite for animal caretakers and visitors [58]. *E*. *histolytica* infection was also found to be prevalent among dogs of younger age group [59]. A report on the molecular detection of *E*. *histolytica/dispar* infection among wild rats in Malaysia corroborates further the risk of zoonotic transmission [60]. Therefore, potential risk of zoonotic agents cannot be ruled out and indicates the importance of developing control measures to prevent transmission by zoonotic mode. To understand the actual dynamics of transmission in North Eastern population of India, genotyping of *E*. *histolytica* strains from humans and animals is highly recommended.

In conclusion, the present study conducted among four North Eastern states showed the highest prevalence rate of *E*. *histolytica* among participants from Assam state. In addition, we have been able to resolve using molecular based techniques, the issue of high rates of microscopically positive samples. The techniques like DNA dot blot hybridization and PCR based detection methods adopted in the present study over and above the conventional screening methods can reduce misdiagnosis of the disease appreciably from the population living in this endemic area. The various logistics associated with the disease that are described here would help the clinicians to better diagnose the patients. Adoption of these diagnostic techniques would help to assess the true epidemiology of this endemic disease prevailing in different parts of India.

## Supporting Information

S1 Table Participant’s demographic profile. (XLS)
